# Supplementary material for: Inhibition of P-Glycoprotein Asymmetrically Alters the In Vivo Exposure Profile of SGC003F: A Novel Guanylate Cyclase Stimulator
Source: Pharmaceuticals (Basel). 2024 Aug 29;17(9):1140. doi: 10.3390/ph17091140 (PMC11435065; doi:10.3390/ph17091140)
Supplement: Supplementary file 1 [file pharmaceuticals-17-01140-s001.zip › pharmaceuticals-3160832-supplementary.pdf]

Supplementary table S1. P_{app} values of SGC003F across LLC-PK1-MDR1, LLC-PK1 MOCK and Caco-2 monolayers (n=3, Mean ± SD).

**Compound**	**LLC-PK1-MOCK**			**LLC-PK1-MDR1**			**Caco-2**		
	**$^{a}P_{app, A-B}$**	**$P_{app, B-A}$**	**ER**	**$P_{app, A-B}$**	**$P_{app, B-A}$**	**ER**	**$P_{app, A-B}$**	**$P_{app, B-A}$**	**ER**
Digoxin	1.49 ± 0.23	1.51 ± 0.21	1.01 ± 0.02	1.13 ± 0.07	11.84 ± 0.89**	10.48 ± 1.48	1.20 ± 0.02	17.40 ± 0.30	14.50 ± 0.06
Digoxin with Tar	1.71 ± 0.20	1.80 ± 0.05	1.05 ± 0.17	5.83 ± 0.74$^{**, \#\#}$	5.28 ± 0.59$^{**, \#\#}$	0.91 ± 0.09	/	/	/
SGC003F	9.07 ± 0.49	9.19 ± 1.38	1.01 ± 0.20	5.27 ± 1.07**	34.55 ± 2.52**	6.56 ± 1.24	5.58 ± 0.56	39.91 ± 2.03	7.15 ± 0.85
SGC003F with Tar	9.61 ± 0.53	10.07 ± 0.82	1.05 ± 0.06	16.24 ± 1.65$^{**, \#\#}$	20.67 ± 0.46$^{**, \#\#}$	1.28 ± 0.12	/	/	/

$^{a}P_{app}$: $\times 10^{-6}$ cm/s. ** $p < 0.01$ (compared to the MOCK group); $^{\#\#}$ $p < 0.01$(compared to the Tar group)

Supplementary table S2. Tissue concentrations and tissue plasma ratio at 0.5 h、1 h、2 h、4 h、24 h of SGC003F in rats with or without oral administration of 15 mg/kg or intravenous administration of 7.5 mg/kg tariquidar (n=3 , Mean ± SD).

		Single		With Tar (*i.v.*)		With Tar (*p.o.*)	
Time point	Matrix	Concentration (ng/mL or ng/g)	K_{P1}	Concentration (ng/mLor ng/g)	K_{P1}	Concentration (ng/mLor ng/g)	K_{P1}
0.5h	Plasma	384±102	/	350±63.5	/	480±101	/
	Heart	188±23.3	0.49±0.07	163±26.9	0.47±0.03	212±40.9	0.44±0.04
	Liver	522±36.3	1.36±0.27	413±77.2	1.19±0.21	459±98.2	0.96±0.14
	Kidney	402±32.1	1.08±0.23	334±22.7	0.97±0.13	419±115	0.87±0.17
	Intestine	577±95.9	1.59±0.52	532±80.1	1.53±0.16	520±125	1.08±0.07
	Brain	2.66±0.83	0.01±0	4.45±3.73	0.01±0.01	4.57±2.18	0.01±0
1h	Plasma	475±78.2	/	753±159	/	828±223	/
	Heart	203±8.84	0.43±0.07	332±122	0.43±0.11	366±110	0.44±0.04
	Liver	553±35.3	1.18±0.17	736±217	0.96±0.11	772±316	0.91±0.19
	Kidney	469±9.15	0.99±0.14	645±161	0.86±0.15	766±104	0.95±0.16
	Intestine	764±497	1.61±1.23	1556±212	2.07±0.26	1146±960	1.31±0.93
	Brain	4.11±1.02	0.01±0	9.91±5.35	0.01±0.01	13.6±7.43	0.02±0.01
2h	Plasma	416±210	/	485±213	/	713±255	/
	Heart	168±66.4	0.42±0.07	222±45.8	0.51±0.18	304±127	0.42±0.03
	Liver	340±144	0.84±0.08	438±164	0.93±0.1	614±251	0.86±0.11

	Kidney	435±104	1.17±0.38	481±157	1.04±0.17	593±252	0.82±0.06
	Intestine	633±208	1.81±0.88	449±2219	0.9±0.08	1244±1165	1.54±0.93
	Brain	3.56±2.01	0.01±0.00	5.33±2.31	0.01±0.01	7.71±4.06	0.01±0.00
4h	Plasma	373±86.1	/	535±106	/	728±163	/
	Heart	169±39.9	0.45±0.04	210±53.6	0.39±0.03	347±153	0.46±0.11
	Liver	335±108	0.88±0.11	440±18.7	0.84±0.15	703±138	0.97±0.09
	Kidney	433±197	1.14±0.31	568±147	1.06±0.16	901±412	1.21±0.33
	Intestine	221.2±36.75	0.59±0.09	311±107	0.57±0.10	669±318	0.92±0.28
	Brain	3.36±1.61	0.01±0.00	10.79±2.15	0.02±0.01	14.1±5.75	0.02±0.00
24h	Plasma	2.95±1.07	/	19.7±13.0	/	28.5±21.6	/
	Heart	0.57±0.41	0.22±0.2	8.03±4.75	0.44±0.06	9.06±9.13	0.27±0.10
	Liver	0.5±0.00	0.18±0.06	11.2±8.04	0.52±0.16	14.0±10.2	0.48±0.11
	Kidney	2.44±1.71	0.77±0.29	25.2±18.3	1.19±0.21	35.0±32.6	1.23±0.37
	Intestine	0.51±0.00	0.18±0.06	10.5±12.1	0.76±0.64	4.97±2.01	0.24±0.13
	Brain	/	/	/	/	/	/

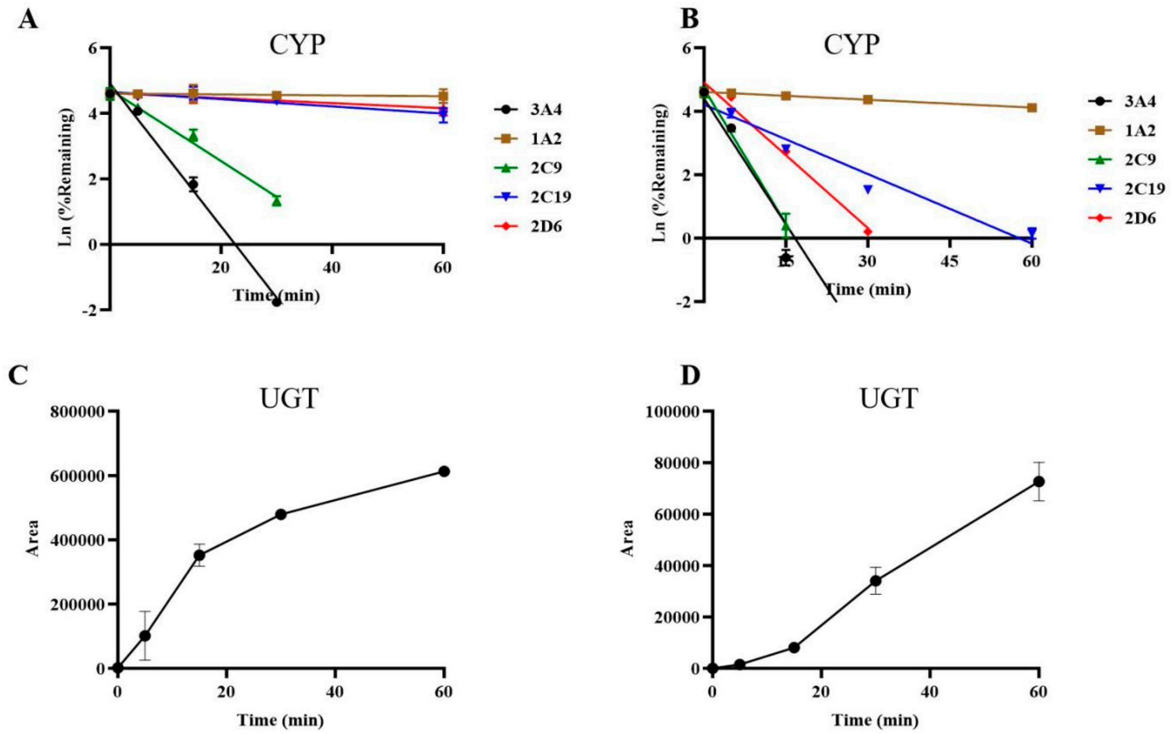

Supplementary Figure S1**.** Metabolic stability of probe substrates in rat (B、D) and human (A、C) liver microsomes. Data are expressed as Mean ± SD. (n=3).
